# Nutrition and listeriosis during pregnancy: a systematic review

**DOI:** 10.1017/jns.2018.16

**Published:** 2018-09-24

**Authors:** L. J. Moran, Y. Verwiel, M. Bahri Khomami, T. J. Roseboom, R. C. Painter

**Affiliations:** 1 Monash Centre for Health Research and Implementation (MCHRI), School of Public Health and Preventive Medicine, Monash University in partnership with Monash Health, 43–51 Kanooka Grove, Clayton, VIC 3168, Australia; 2 Department of Obstetrics and Gynecology, Academic Medical Center, University of Amsterdam, Amsterdam, The Netherlands

**Keywords:** Listeriosis, *Listeria monocytogenes*, Pregnancy, Food

## Abstract

Listeriosis is a rare but severe foodborne illness which is more common in populations such as pregnant women, and can result in serious complications including miscarriage, prematurity, maternal and neonatal sepsis, and death in the newborn. Population recommendations exist for specific foods and food preparation practices to reduce listeriosis risk during pregnancy. The aim of the present systematic review was to assess the association between listeriosis and these practices during pregnancy to confirm appropriateness of these recommendations. We searched MEDLINE, Embase, CINAHL Plus, Web of Science Core Collection, included articles’ references, and contacted clinical experts. All databases were searched until July 2017. Case–control and cohort studies were included which assessed pregnant women or their newborn offspring with known listeriosis status and a nutritional exposure consistent with international population recommendations for minimising listeriosis. Outcomes included listeriosis with or without pregnancy outcomes. Risk of bias was assessed through the Newcastle–Ottawa Scale. Results were described narratively due to clinical heterogeneity in differences in nutritional exposures. Eleven articles comprising case–control or cross-sectional studies met the inclusion criteria. Cases of maternal, fetal or neonate listeriosis were more likely to have consumed high-risk dairy products, meat products or some fruits during pregnancy in comparison with women without listeriosis. Cases of listeriosis were more likely to have consumed foods that are highlighted in population guidelines to avoid to minimise listeriosis in comparison with those without listeriosis during pregnancy. Further research is warranted assessing means of improving the reach, uptake and generalisability of population guidelines for reducing listeriosis during pregnancy.

*Listeria monocytogenes* is a foodborne pathogen which causes listeriosis, a systemic illness that can cause symptoms ranging from gastroenteritis to meningitis and severe sepsis. *L. monocytogenes* is environmentally widespread and able to contaminate a range of foods^(^^1^^)^, accounting for 19 and 17 % of the known causes of foodborne disease-related deaths in the USA and France, respectively^(^^2^^,^^3^^)^. Overall, listeriosis was estimated to have caused 23 150 illnesses, 5463 deaths and 172 823 disability-adjusted life years worldwide in 2010^(^^4^^)^. *L. monocytogenes* is relatively resistant to diverse environments, which enables it to survive food processing and grow in refrigerated or ready-to-eat chilled foods^(^^5^^)^. It is therefore difficult to control in the food production environment, and consequently listeriosis may arise from food contamination outbreaks or from sporadic consumption of contaminated food^(^^6^^,^^7^^)^. Recommendations to avoid listeriosis differ slightly between countries but generally include avoiding high-risk foods which are susceptible to *L. monocytogenes* contamination, such as pre-prepared or pre-packaged salads, pre-prepared foods, soft, semi-soft and surface-ripened cheeses, processed meats and unpasteurised dairy products, and practising appropriate food hygiene practices^(^^8^^,^^9^^)^.

Groups at higher risk of contracting listeriosis include the immunocompromised, the elderly, pregnant women and neonates. Pregnant women appear 10–20 times more likely to contract listeriosis^(^^1^^)^ compared with the general population, possibly owing to a down-regulation of cellular immunity^(^^1^^)^. Listeriosis in pregnancy is generally defined as a clinical illness in a mother and/or child in conjunction with isolation of *L. monocytogenes* from the mother, neonate, fetus or placental surface^(^^10^^)^.

Listeriosis can frequently affect the fetus and neonate by transplacental transmission^(^^1^^)^. Pregnancy-related cases account for 20·7 % of listeriosis globally, with an overall case fatality of 14·9 %, including neonatal deaths^(^^4^^)^. Mothers with listeriosis may be asymptomatic or have influenza-like symptoms such as fever, malaise, myalgia or headache^(^^1^^,^^7^^,^^11^^)^. Listeriosis can result in more serious consequences in the fetus or neonate, including miscarriage, prematurity, central nervous system infections, septicaemia and death^(^^1^^,^^4^^,^^12^^)^ due to immune system insufficiency.

The incidence of listeriosis during pregnancy has decreased recently in a number of countries, including France, Belgium and the USA^(^^12^^–^^14^^)^, possibly due to regulatory and industry efforts, or increased awareness following active prevention campaigns targeting pregnant women^(^^12^^,^^13^^)^. However, it may be under-reported due to variable or asymptomatic maternal presentation or under-recognition in spontaneous miscarriages or stillbirths. It is thus still important to minimise the risk of development of listeriosis during pregnancy due to the severity of the implications for fetal and neonatal health.

Recommendations for reducing nutritional exposure to high-risk or contaminated foods are the best ways to reduce the risk of listeriosis. The objective of the present systematic review was therefore to assess the association between foods and food preparation practices and listeriosis during pregnancy to guide the refinement of population-specific recommendations.

## Methods

### Eligibility criteria

We included case–control and cohort studies. Case reports, case series and reviews were excluded. Eligible studies included pregnant women or their newborn offspring with known listeriosis status. Exclusion criteria were reports on men or non-pregnant women and animal studies. Only articles published in English were included. The protocol was registered in the international prospective register of systematic reviews PROSPERO (CRD 42017056134). No ethics or institutional review board approval was required for this work.

The aetiological exposure was defined as nutritional exposure during pregnancy consistent with international population recommendations (such as Food Standards Australia New Zealand or the Centers for Disease Control and Prevention) for minimising listeriosis, including delicatessen-style meat, cold meats, dairy products made from unpasteurised milk, soft, semi-soft or surface-ripened cheeses, ready-to-eat foods, and appropriate food preparation and storage techniques^(^^8^^,^^9^^)^.

The primary outcome was a diagnosis of listeriosis during pregnancy. Secondary outcomes were maternal (pregnancy complications including miscarriage, preterm delivery, maternal sepsis, chorioamnionitis or hospitalisation), fetal (stillbirth) or neonatal (death, preterm birth, admission to hospital or infection) outcomes.

### Information sources and search strategy

A comprehensive database search was conducted on 6 July 2017 to identify all articles published prior to this date. The following electronic databases were used to identify relevant published literature: MEDLINE in-process and other non-indexed citations (Ovid MEDLINE^(R)^ In-Process & Other Non-Indexed Citations, Ovid MEDLINE^(R)^ Daily and Ovid MEDLINE^(R)^ 1946 to Present), Embase (Embase Classic + Embase 1947 to 3 July 2017), CINAHL Plus and Web of Science Core Collection. The search strategy for MEDLINE is documented in Supplementary Table S1 and was modified for the other databases using relevant subject headings. Additional eligible articles were identified by hand-searching the reference lists of all included articles, or by contacting clinical experts. Where abstracts were identified, these were included if a corresponding full-text article could be identified.

### Study selection

One independent reviewer (L. J. M.) who was not blinded to the names of investigators or sources of publication, identified and selected the articles that met the inclusion criteria. A second independent reviewer (Y. V.) performed article selection on a subset (10 %) of articles. Disagreements between these two authors were discussed and resolved by consensus or arbitration.

### Data extraction

Relevant data from included studies were extracted independently by one reviewer (L. J. M.), with a second reviewer (M. B. K.) independently checking data extraction for all of the eligible studies. Disagreements between these two authors were discussed and resolved by consensus or arbitration. The data extracted included information on author(s), year of publication, study design, study location, participant characteristics, nutritional exposures (retrospective or prospective) and outcomes.

### Assessment of risk of bias

All included studies were evaluated by one independent reviewer (L. J. M.), with a second reviewer (M. B. K.) independently evaluating all of the eligible studies, with neither of the two authors blinded to the names of investigators or sources of publication. The quality of the included studies was assessed using criteria based on the Newcastle–Ottawa Scale for non-randomised studies, with a maximum score of 9^(^^15^^)^. These criteria assess the selection of case and control groups, comparability of case and control groups, and the quality of outcome measurement. Studies were classified as good quality if they scored 3 or 4 in the selection domain, 1 or 2 in the comparability domain, and 2 or 3 in the outcome/exposure domain; fair quality if they scored 2 in the selection domain, 1 or 2 in the comparability domain, and 2 or 3 in the outcome/exposure domain; and poor quality if they scored 0 or 1 in the selection domain, or 0 in the comparability domain, or 0 or 1 in the outcome/exposure domain.

### Data synthesis

Data were presented as subgroups of (1) studies where data were available for pregnant women and neonates as a separate group and (2) studies where data were not available for pregnant women and infants as a separate group but instead were presented as combined data. Due to clinical heterogeneity relating to differences in nutritional exposures, it was not possible to perform a meta-analysis, and thus results are described narratively. It was not possible to assess publication bias through funnel plots as no statistical data synthesis could be performed.

## Results

### Study selection

**Fig. 1.**
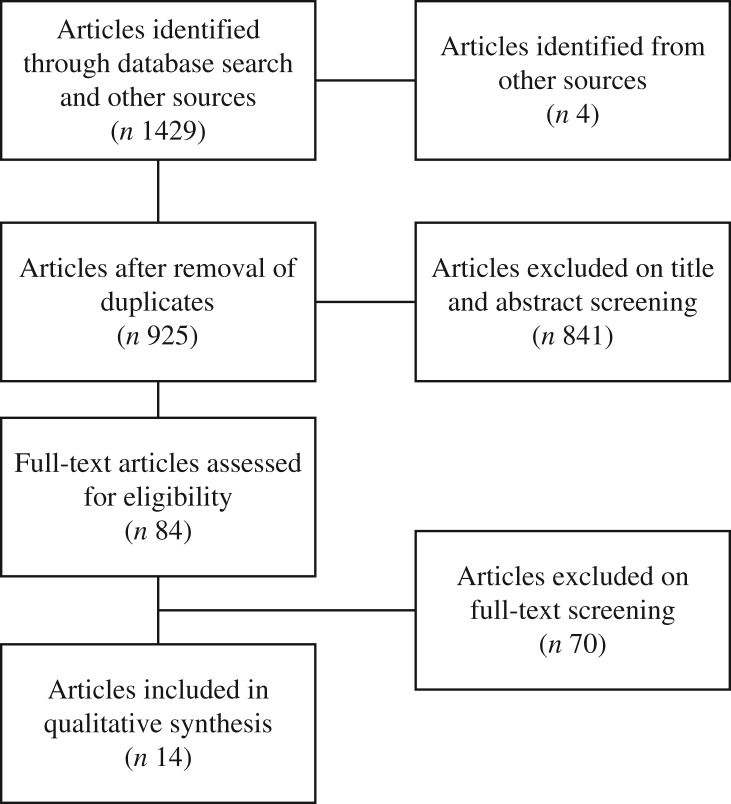
Flow chart of article selection.

The database searches yielded 1429 citations, with an additional four articles identified from clinical experts. After removal of duplicates, 925 citations remained. On the basis of *a priori* selection criteria, screening of titles or abstracts resulted in eighty-four papers being identified for full-text assessment. Of these, seventy articles were excluded, with reasons for exclusion detailed in Supplementary Table S2. We included fourteen full-text articles (comprising eleven studies) for our final analysis (Fig. 1)^(^^6^^,^^10^^,^^16^^–^^27^^)^.

### Study characteristics

The characteristics of included studies are presented in Table 1 and Supplementary Table S3. The studies were all case–control studies, with the exception of one cross-sectional study^(^^23^^)^. The studies were conducted in the USA^(^^6^^,^^21^^,^^22^^,^^24^^–^^27^^)^, Denmark^(^^20^^)^, France^(^^19^^)^, Australia^(^^10^^)^ and Iran^(^^23^^)^. Five studies assessed outbreak cases^(^^6^^,^^19^^,^^21^^,^^22^^,^^24^^)^, five studies assessed non-outbreak cases^(^^10^^,^^23^^,^^25^^–^^27^^)^, and one study assessed both outbreak and non-outbreak cases^(^^20^^)^ of listeriosis. The study sample sizes varied across included studies, from *n* 55^(^^22^^)^ to *n* 545^(^^26^^)^. Where documented specifically for the cases of listeriosis during pregnancy, the participant age ranged from 16 to 40 years^(^^19^^,^^21^^–^^23^^,^^26^^)^. Ethnicity data were provided for a minority of studies. Of these, the majority of the study population was Caucasian^(^^26^^)^, Hispanic^(^^21^^,^^22^^)^, Iranian^(^^23^^)^ or European^(^^19^^)^.

**Table 1.** Characteristics of included studies

Author and year	Country	Study type	Time study conducted	Outbreak or non-outbreak	Number	Collection of information on dietary intake	Study quality
Schlech 1983^(^^24^^)^	USA, Canada	Case–control	January 1971–June 1981	Outbreak	Total: 205 (41 cases, 164 controls) Perinatal: 34 cases, unclear controls but assume 4 matched	General food history 3 months before onset of illness Telephone or in-person interview	Fair quality
Fleming 1985^(^^6^^)^	USA	Case–control	January 1982–August 1983	Outbreak	Total: study 1, 57 (19 cases, 38 controls) Study 2, 80 (40 cases, 40 controls) Perinatal: unclear. 7/49 of original outbreak cases perinatal	Not stated	Fair quality
Schwartz 1988^(^^25^^)^	USA	Case–control	September 1986–July 1987	Non-outbreak	Total: 321 (82 cases, 239 controls) Perinatal: 26 cases, unclear controls but assume 4 matched	Diet history including food items associated with listeriosis or other foodborne bacterial diseases (including raw fruits and vegetables, meat and poultry, eggs, dairy products, processed and pickled meat) 1 month before date of positive culture Interview	Good quality
Linnan 1988^(^^21^^)^	USA	Case–control	January 1985–May 1987	Outbreak	All perinatal: 78 (39 cases, 39 controls)	Assessment of more than 60 food items Means of assessment not stated	Good quality
Schuchat 1992^(^^27^^)^	USA	Case–control	November 1988–December 1990	Non-outbreak	Total: 541 (165 cases, 376 controls) Perinatal: 67 cases, unclear controls but assume 2·3 matched	Exposure to specific foods and food preparation methods 1 month before illness onset Interviews	Good quality
Jensen 1994^(^^20^^)^	Denmark	Case–control	January 1989–May 1991	Outbreak and non-outbreak	Total: 90 (50 cases, 40 controls) Perinatal: unclear, assume from surveillance characteristics. 10 (10 %) of all cases materno-fetal	More than 50 food items 1 month before date of positive specimen Questionnaire	Fair quality
Goulet 1998^(^^19^^)^	France	Case–control	June 1993–October 1993	Outbreak	Total: 98 (21 cases, 77 controls) Perinatal: 22 cases, unclear controls but assume 4 matched	>100 Food items associated with listeriosis (mainly dairy/meat products) and brand names, type of packaging, frequency of consumption and food store purchased from 1 month before onset of illness Telephone interview, standardised questionnaire	Fair quality
MacDonald 2005^(^^22^^)^	USA	Case–control	October 2000–January 2001	Outbreak	All perinatal: 55 (11 cases, 44 controls)	46 Food items and shopping histories 1 month before illness developed Standardised questionnaire	Good quality
Varma 2007^(^^26^^)^	USA	Case–control	2000–2003	Non-outbreak	Total: 545 (169 cases, 376 controls) Perinatal: 28 cases, unclear controls but assume 4 matched	>100 Food and drink items and where they were prepared or consumed 1 month before specimen collection Standardised telephone interviews	Fair quality
Dalton 2011^(^^10^^)^	Australia	Case–control	November 2001–December 2004	Non-outbreak	Total: 233 (136 cases, 97 controls) Perinatal: 31 (19 cases, 12 controls)	43 foods considered to be high risk for listeriosis: frequency of consumption, place of preparation, site of purchase or consumption and consumed raw/cooked 1 month prior to specimen collection Telephone interviews, standardised questionnaire	Poor quality
Pourkaveh 2016^(^^23^^)^	Iran	Cross-sectional	January 2015–December 2015	Non-outbreak	All perinatal: 317 (54 cases, 263 controls)	Risk factors for listeriosis including unpasteurised dairy products; feta or soft cheese; raw, half baked, processed or smoked meat; raw or ready-to-eat vegetables; raw or smoked seafood 3 months before and during pregnancy Face-to-face interview, questionnaire developed by authors	Poor quality

Information on dietary exposures was collected by various methods, including a general food history^(^^24^^)^ with an additional assessment of specific food items or food preparation methods implicated with *L. monocytogenes* or other foodborne diseases^(^^10^^,^^19^^–^^23^^,^^26^^,^^27^^)^. Information was collected through methods including questionnaires, or telephone or face-to-face interviews. In all studies, nutritional information was assessed retrospectively from 1 month^(^^10^^,^^19^^,^^20^^,^^22^^,^^25^^–^^27^^)^ to 3 months^(^^23^^,^^24^^)^ prior to illness onset or positive culture.

Listeriosis cases were identified from medical practitioners^(^^21^^,^^25^^,^^27^^)^, hospital admissions^(^^22^^–^^24^^)^, medical records^(^^21^^,^^25^^,^^27^^)^, microbiological laboratories^(^^6^^,^^10^^,^^19^^–^^22^^,^^26^^,^^27^^)^ and health departments^(^^21^^,^^26^^)^. Controls were identified from sources including being at the same hospital^(^^10^^,^^19^^–^^21^^,^^23^^,^^24^^,^^26^^)^, town or county^(^^6^^,^^22^^)^ or medical practitioner^(^^6^^,^^19^^,^^25^^–^^27^^)^. Listeriosis during pregnancy was diagnosed as a clinical illness in a mother and/or child and/or isolation of *L. monocytogenes* from the mother, neonate, fetus or placental surface^(^^6^^,^^10^^,^^19^^–^^24^^,^^26^^,^^27^^)^. Specific criteria for each study included a positive culture or isolation of *L. monocytogenes* from mother, fetus or neonate^(^^6^^,^^10^^,^^19^^,^^21^^–^^24^^,^^27^^)^, and/or illness or death in a mother and/or child^(^^6^^,^^10^^,^^20^^,^^22^^,^^24^^,^^26^^)^, or no specific details provided^(^^25^^)^.

### Risk of bias of included studies

The quality assessment of the included studies is presented in Table 1 and Supplementary Table S4. Overall, four studies were classified as good quality^(^^21^^,^^22^^,^^25^^,^^27^^)^, five studies were classified as fair quality^(^^6^^,^^19^^,^^20^^,^^24^^,^^26^^)^ and two studies were classified as poor quality^(^^10^^,^^23^^)^. The case definition was adequate and was independently validated for all studies. The representativeness of the cases was adequate with the exception of one study for which it was not stated if the cases comprised all eligible cases in a certain catchment area or sample of these cases^(^^23^^)^, and three studies for which the surveillance population was representative of the broader population but it was not stated how the cases were selected for the case–control study from this population^(^^6^^,^^19^^,^^24^^)^. The selection of the controls was adequate for all studies, with the exception of three studies where hospital controls were used^(^^20^^,^^23^^,^^26^^)^. None of the studies stated that the controls had no history of listeriosis. For the comparability of the cases and controls on the basis of design or analysis, four studies were comparable on one factor^(^^19^^,^^24^^–^^26^^)^, and six studies^(^^6^^,^^10^^,^^20^^–^^22^^,^^27^^)^ were comparable on two or more factors. The cases and controls were matched for characteristics including time of birth^(^^24^^)^, birth weight^(^^24^^)^, gestational age^(^^19^^,^^21^^,^^22^^,^^26^^,^^27^^)^, maternal age^(^^6^^,^^10^^,^^20^^–^^22^^,^^25^^,^^27^^)^, geographical area^(^^6^^,^^20^^)^, hospital^(^^19^^,^^21^^)^, ethnicity^(^^21^^,^^22^^)^ and health-care provider^(^^27^^)^. Pourkaveh *et al*.^(^^23^^)^ did not perform matching for any study characteristics and noted that cases were younger and were less likely to have a tertiary qualification or Internet access. The ascertainment of exposure was performed by a structured interview, where the outcome assessor was blinded to case or control status for three studies^(^^24^^,^^25^^,^^27^^)^, an interviewer where the outcome assessor was not blinded to case or control status for five studies^(^^10^^,^^19^^,^^20^^,^^22^^,^^23^^,^^26^^)^, and no description for two studies^(^^6^^,^^21^^)^. The exposure was ascertained in the same way for cases and controls for all studies. The non-response rate was the same for cases and controls, with the exception of one study for which *n* 5 were missing from non-pregnancy cases^(^^10^^)^, and one study where non-respondents were described^(^^23^^)^.

### Synthesis of results

#### Nutritional exposure

The dietary intake data for the included studies are presented in Table 2. Four studies reported nutritional intake data for the cases of listeriosis during pregnancy subset separately, of which two were outbreak^(^^21^^,^^22^^)^ and two were non-outbreak^(^^10^^,^^23^^)^. Cases were more likely to have consumed dairy products (unpasteurised dairy products^(^^20^^)^, soft cheeses^(^^23^^)^ or Mexican-style cheeses^(^^21^^,^^22^^)^), meat products (hot dogs^(^^22^^)^, semi-cooked, smoked or processed meat or smoked seafood^(^^23^^)^) or vegetables (ready-to-eat vegetables^(^^23^^)^ or *jicama* (root vegetable)^(^^21^^)^). The OR for listeriosis during pregnancy after consumption of these foods ranged from 4·12 to 17·8. Conversely, there were no significant associations reported between the consumption of the following foods: rockmelon/cantaloupe, ready-to-eat fruit salad, ready-to-eat other salad, chopped liver/liverwurst, Camembert, blue-veined cheese, feta, mussels and listeriosis in one study^(^^10^^)^.

**Table 2.** Dietary intake and maternal, neonatal and infant outcomes

Author	Dietary intake	Secondary maternal, fetal or neonatal outcomes
Schlech 1983^(^^24^^)^	Cases: more likely consumed coleslaw (OR 2·31; *P* = 0·04) on multivariate analysis	Cases (perinatal): *n* 5 acute febrile illness and spontaneous abortion, *n* 4 stillbirth, *n* 23 live birth of seriously ill premature or term infant, *n* 2 live birth of well infant, 27 % case-fatality rate for infants born alive
Fleming 1985^(^^6^^)^	Study 1: no association with consumption of meats, fresh vegetables, coleslaw and other salads or unpasteurised dairy products and listeriosis. Association between chain A whole or 2 % pasteurised milk (OR 9·3; *P* < 0·01) Study 2: association between chain A whole or 2 % pasteurised milk (OR 11·5; 95 % CI 2·7, 48·8; *P* < 0·001)	Cases (perinatal): *n* 2 death *in utero*, *n* 3 meningitis, *n* 2 septicaemia Case fatality: 29 %
Schwartz 1988^(^^25^^)^	Cases: more likely eaten uncooked hot dogs (OR 12·3; 95 % CI 1·6, 97·3) or undercooked chicken (OR 20·5; 95 % CI 1·2, 343) on multivariate analysis	Case fatality (perinatal and non-perinatal): 22 %
Linnan 1988^(^^21^^)^	Cases: more likely consumed Mexican-style cheeses (OR 5·5; 95 % CI 1·2, 24·8) and *jicama* (root vegetable) (OR 4·12; 95 % CI 1·2, 13·2)	Cases (perinatal from larger surveillance study): Sepsis – 88 % (early fetal/neonatal); 17 % (late fetal/neonatal); 52 % (maternal) Meningitis – 2 % (early fetal/neonatal); 67 % (late fetal/neonatal); 0 % (maternal) Sepsis and meningitis – 6 % (early fetal/neonatal); 17 % (late fetal/neonatal); 0 (maternal), *n* 10 neonatal deaths, *n* 20 fetal deaths, *n* 0 maternal deaths Case fatality: early fetal/neonatal 63 %, late fetal/neonatal 0 %
Schuchat 1992^(^^27^^)^	Cases: more likely consumed soft cheeses (OR 2·6; 95 % CI 1·4, 4·8; *P* = 0·002) or food purchased from store delicatessen counters (OR 1·6; 95 % CI 1·0, 2·5; *P* = 0·04) and less likely to have consumed macaroni salad (OR 0·48; 95 % CI 0·30, 0·77; *P* = 0·002) and raw apples (OR 0·51; 95 % CI 0·33, 0·81; *P* = 0·004) on multivariate analysis 32 % of sporadic disease attributed to eating soft cheeses and food purchases from store delicatessen counters	No details
Jensen 1994^(^^20^^)^	Cases: More likely consumed pâté (OR 8·1; *P* < 0·07), unpasteurised milk (OR 2·4; *P* > 0·1) or pasteurised milk (OR 2·4; *P* > 0·1)	No details
Goulet 1998^(^^19^^)^	Cases: more likely consumed rillettes (pork product) (OR 10·9; 95 % CI 2·1, 54·4) or country pâté (OR 5·0; 95 % CI 1·0, 24·1) from brand A store on multivariate analysis and same can of rillettes during several meals (>4 meals, 11/23 *v.* 7/31; *P* = 0·05)	Cases (perinatal): *n* 9 fetal deaths, *n* 12 premature births (*n* 5 severe prematurity), *n* 10 term births, duration of hospitalisation of surviving neonates (mean 14 d, range 5–76 d), all except 2 mothers had 1 or more symptoms or clinical signs (*n* 29 fever, *n* 15 chills, *n* 14 headache myalgia, *n* 1 diarrhoea) Case fatality: 32 %
MacDonald 2005^(^^22^^)^	Cases: more likely consumed fresh, unlabelled, Mexican-style cheese sold by door-to-door vendors (OR 17·5; 95 % CI 2·0, 152·5); *queso fresco*, a Mexican-style soft cheese (OR 7·3; 95 % CI, 1·4, 37·5), *queso ranchero*, another Mexican-style fresh cheese (OR 9·5; 95 % CI 1·8, 50·0) and hot dogs (OR 4·6; 95 % CI, 1·1, 19·4) on univariable analysis and combined cheese (*queso ranchero* and *queso fresco*) (OR 17·8; 95 % CI 1·9, 169·6) on bivariate analysis	Cases (perinatal): *n* 5 stillbirths, *n* 3 premature deliveries, *n* 3 infected newborns Symptoms prior to hospital admission fever (83 %), chills (83 %), headache (83 %), abdominal cramps (50 %), stiff neck (50 %), vomiting (25 %), photophobia (25 %)
Varma 2007^(^^26^^)^	Cases: more likely consumed melons at commercial establishment (OR 2·6; 95 % CI 1·4, 5·0; *P* = 0·01) and hummus prepared in commercial establishment (OR 5·7; 95 % CI 1·7, 19·1; *P* < 0·01) on multivariable analysis	Cases (perinatal): 64 % hospitalised for *Listeria monocytogenes*-associated illness (median 8 nights; range 2–9 nights), *n* 7 (28 %) spontaneous abortion or fetal demise (fetal loss at median 18 weeks, range 16–35 weeks), 15/18 infants from live births hospitalised after delivery for median of 10 nights (range 1–54 nights)
Dalton 2011^(^^10^^)^	Perinatal data: no association between listeriosis and any foods (rockmelon/cantaloupe, ready-to-eat fruit or other salad, self-serve salad bar, lettuce, prepacked bagged coleslaw, alfalfa/pea sprouts, ready-to-eat coleslaw, chopped liver/liverwurst, Camembert, blue-veined cheese, feta, mussels)	Cases (perinatal): *n* 4 fetal deaths Case fatality (fetal): 21 %
Pourkaveh 2016^(^^23^^)^	Cases: higher consumption of unpasteurised dairy products (68·5 *v.* 0·8 %), soft cheese (74·1 *v.* 4·2 %), semi-cooked (46·3 *v.* 1·9 %) or smoked (33·3 *v.* 1·6 %) meat, processed meat (50 *v.* 0·4 %), smoked seafood (22·2 *v.* 8·4 %) and ready-to-eat vegetables (raw or unwashed) (31·5 *v.* 1·9 %) during pregnancy (all *P* < 0·001)	Cases (perinatal): 69·4 % complete spontaneous abortion, 100 % vaginal discharge, 96·3 % lower abdominal pain Cases and controls: no difference in gestational age of second or third spontaneous abortion for cases *v.* controls. Gestational age of current abortion (8·6/54 *v.* 10·1/263; *P* < 0·001) and first spontaneous abortion (8·6/54 *v.* 9·9/263; *P* < 0·001) lower for cases *v.* controls

For the studies reporting combined data for listeriosis during pregnancy and listeriosis not during pregnancy, three studies assessed outbreak cases^(^^6^^,^^19^^,^^24^^)^, three studies assessed non-outbreak cases^(^^25^^–^^27^^)^, and one study assessed both outbreak and non-outbreak cases^(^^20^^)^ of listeriosis. Cases were more likely to have consumed dairy products (pasteurised milk^(^^6^^,^^20^^)^, unpasteurised milk^(^^20^^)^ or soft cheeses^(^^27^^)^), meat products (uncooked hot dogs, undercooked chicken^(^^25^^)^, rillettes (pork product)^(^^19^^)^ or pâté^(^^19^^,^^20^^)^), fruits or vegetables (melons and hummus prepared at a commercial establishment^(^^26^^)^ or coleslaw^(^^24^^)^) or food purchased from store delicatessen counters^(^^27^^)^. The OR for listeriosis after consumption of these foods ranged from 1·6 to 20·5. Cases were also less likely to have consumed macaroni salad and raw apples^(^^27^^)^. Only one study assessed food preparation methods and no data were reported for this outcome^(^^27^^)^.

#### Maternal, fetal and neonatal outcomes

Secondary maternal, fetal and neonatal outcomes are reported in Table 2. These were only reported for the cases for the majority of studies^(^^6^^,^^10^^,^^19^^,^^20^^,^^21^^,^^22^^,^^24^^,^^26^^)^. Reported health outcomes included acute febrile illness, diarrhoea, abdominal cramps, stiff neck, vomiting, photophobia, headache, spontaneous abortion, stillbirth, premature birth, live birth of a seriously ill premature or term neonate, *in utero* death, meningitis and septicaemia^(^^6^^,^^19^^,^^21^^,^^22^^,^^24^^)^. Only one study reported outcomes (spontaneous miscarriage and gestational age at miscarriage) separately for both cases and controls^(^^23^^)^. The gestational age of the current and the first spontaneous miscarriage was lower for the women with listeriosis compared with those without listeriosis.

## Discussion

We report here a systematic review assessing the contribution of specific foods to listeriosis in pregnancy. Both outbreak and non-outbreak studies included in the present systematic review report that mothers with listeriosis during pregnancy were more likely to have consumed either pasteurised or unpasteurised dairy products, cooked, semi-cooked, smoked or processed meat products, or ready-to-eat foods including fruits and vegetables, although we note the OR for an increased consumption of these foods ranged from 1·6 to 20·5. With regards to secondary maternal, fetal and neonatal outcomes, these ranged from acute febrile illness and gastrointestinal discomfort to premature birth, meningitis, septicaemia and neonatal mortality. Only one study reported secondary maternal, fetal and neonatal outcomes separately for both cases and controls, and reported a lower gestational age of the current and first spontaneous abortion for cases compared with controls^(^^23^^)^.

These findings support guidelines for preventing listeriosis during pregnancy. The results showed an increased risk for listeriosis during pregnancy due to the consumption of a number of dairy food products, including unpasteurised products such as raw milk, soft cheeses or Hispanic-style cheeses. These are high-risk food products for pregnant women as they either do not undergo a treatment process to remove *L. monocytogenes*, or *L. monocytogenes* can survive the production process^(^^28^^)^. Furthermore, two studies comprised outbreaks associated with the consumption of Mexican-style cheese. These styles of cheeses mainly infect pregnant women with a Hispanic ethnicity in US studies^(^^21^^,^^22^^)^ and are high-risk foods for listeriosis due to the fact that they are manufactured from raw milk, undergoing processing by thermoplastification or ripening which is insufficient to kill foodborne pathogens or contamination at the post-processing stage^(^^29^^)^. Population recommendations should include culturally relevant information on specific higher-risk foods to avoid and safer food alternatives, in addition to safe food preparation methods^(^^30^^)^. This is particularly important as there may be a lower uptake or delivery of food safety messages to ethnic minorities^(^^31^^)^ and higher pregnancy-associated listeriosis in ethnic minorities^(^^32^^,^^33^^)^.

Outbreaks of listeriosis related to the consumption of pasteurised products can occur due to contamination after the heat-treatment process^(^^34^^)^. However, these result in fewer hospitalisations than outbreaks caused by unpasteurised products^(^^34^^)^. An association between listeriosis and pasteurised products was observed here in two studies^(^^6^^,^^20^^)^, although we note that these were comprised predominantly of outbreak cases and that the association reported by Jensen *et al*.^(^^20^^)^ was of borderline statistical significance. This suggests that guidelines for reducing listeriosis risk need not be modified to incorporate pasteurised products. Our results also showed an association between consumption of ready-to-eat products or products prepared in commercial establishments and a range of meat products and a higher risk of listeriosis in pregnancy including uncooked, undercooked, processed or smoked meats or pâté or smoked fish^(^^19^^,^^20^^,^^22^^–^^27^^)^. These products are high risk as *L. monocytogenes* can grow and multiply during cold storage^(^^35^^)^. As these foods are often consumed without further heating, this will increase the chance of a consumer being affected by a contaminated product. This is consistent with ready-to-eat products such as delicatessen meats being classified in the very high-risk category for potential risk of listeriosis^(^^36^^)^. This highlights the importance of physical facility, equipment design and cross-contamination controls to avoid contamination of products, and of outbreak investigations in identifying suspected food vehicles for foodborne illness.

While dietary guidelines for preventing listeriosis differ from country to country, in general they consistently cover the themes of specific high-risk foods and food handling practices to avoid^(^^8^^,^^9^^)^. The dietary exposures associated with listeriosis in the present systematic review were in keeping with these dietary guidelines in that unpasteurised dairy products, soft cheeses, undercooked, uncooked, smoked or processed meat, and ready-to-eat foods were associated with a higher risk of listeriosis. This highlights the utility of the current guidelines. However, there is some concern that these guidelines may overly focus on exclusion of certain foods and handling practices. It is possible that the lack of a focus on the inclusion of safer alternatives and practices may overall lead to poorer diet quality^(^^8^^,^^9^^)^. The uptake of the current recommendations in different populations is also unknown given variations in health literacy with demographic characteristics such as age and education^(^^37^^,^^38^^)^. This is consistent both with our finding of younger age and lower education and Internet access for cases compared with controls^(^^23^^)^. It is also consistent with previous reports of an association of listeriosis with lower age^(^^39^^)^ or lower socio-economic status^(^^40^^)^, which is also of concern given potential delays in appropriate specimen collection to confirm illness for individuals living in more deprived areas^(^^41^^)^.

While we did not include literature reporting on listeriosis outbreaks more broadly, these were beyond the scope of the present review inclusion criteria. These focused solely on case–control and cohort studies to allow a comparison of nutritional exposures in cases compared with controls. We note important findings from the broader listeriosis outbreak literature including confirmation of high-risk foods reported here such as soft cheeses^(^^42^^)^, smoked fish^(^^43^^)^, processed meats^(^^44^^)^, ready-to-eat vegetables^(^^45^^,^^46^^)^ and pâté^(^^47^^)^. We also highlight that this broader outbreak literature aids in identification of high-risk foods that were not significantly associated with listeriosis in the limited identified literature on case–control and cohort studies in pregnancy, such as rockmelon or cantaloupe^(^^48^^)^. These foods are well documented as being associated with outbreaks in the past which have included pregnant women^(^^48^^)^. Where foods identified as high risk from population outbreaks such as vegetables^(^^45^^,^^46^^)^ were not significantly associated with listeriosis here^(^^10^^)^, the authors also comment on potential limited power to detect significant associations. This supports the inclusion of these foods in current population recommendations for minimising listeriosis^(^^8^^)^.

We identified a relatively limited literature base of case–control studies examining the association between specific foods and listeriosis, and an even smaller number of studies that reported data for the cases during pregnancy separately^(^^10^^,^^21^^–^^23^^)^. Furthermore, the majority of studies reported on maternal, fetal and neonatal complications associated with listeriosis just for the cases, with only one study reporting on differences in perinatal complications between cases and controls^(^^23^^)^. This made it impossible for us to quantify the effects of consumption of various foods in pregnancy on adverse pregnancy outcomes mediated by listeriosis. We note that many exposures are required to cause one case of pregnancy-associated listeriosis (estimated at about 1 per 10 000 exposed pregnant women)^(^^49^^)^, which may limit the utility of case–control studies in assessing the relationship between outcome and exposures. Additional limitations relating to the individual studies include the possibility of recall bias due to the studies being case–control and also as dietary information was retrospectively collected 1–3 months prior to disease confirmation in all studies. The shorter time-frame may also have contributed to bias as the incubation period in pregnancy-associated listeriosis cases ranges from 17 to 67 d^(^^50^^)^. Dietary information was collected by a blinded investigator in only a limited number of studies^(^^24^^,^^25^^,^^27^^)^, which increases the possibility of detection bias. We also note variability in the source and matching of controls, and heterogeneity in the studies in that they comprised both outbreak and non-outbreak cases of listeriosis, which makes it difficult to interpret data more broadly for general background risk of listeriosis. The bulk of the literature (seven out of eleven studies) also focused on studies from the USA; thus research from a broader range of countries is warranted to maximise generalisability of these results. The studies also generally examined only specific food consumption, with a more limited number examining different means of food preparation^(^^27^^)^, and no studies examining food hygiene practices. As all of these aspects are highlighted in recommendations for reducing listeriosis^(^^8^^,^^9^^)^, they also warrant assessment in future studies. Additional biases may also have occurred through identifying articles only in English.

Listeriosis is associated with severe maternal, fetal and neonatal complications and warrants consumer and health professional guidelines to minimise risk. In the present systematic review, cases of listeriosis during pregnancy were more likely to have consumed certain high-risk dairy and meat products and ready-to-eat foods in comparison with controls, with avoidance of these specific high-risk foods generally being recommended in population guidelines. Further research is warranted assessing the reach and uptake of population guidelines for preventing listeriosis in pregnant women from a range of countries, reproductive life stages and demographic backgrounds. This will aid in increasing the uptake of guidelines to reduce cases of listeriosis during pregnancy and materno-fetal morbidity and mortality.

## Supplementary material

For supplementary material accompanying this paper visit https://doi.org/10.1017/jns.2018.16. click here to view supplementary material
