# Supplementary material for: Nutrition and listeriosis during pregnancy: a systematic review
Source: J Nutr Sci. 2018 Sep 24;7:e25. doi: 10.1017/jns.2018.16 (PMC6161013; doi:10.1017/jns.2018.16)
Supplement: Supplementary file 1 [file S2048679018000162sup001.docx]

**Supplementary Table S1.** Search strategy in MEDLINE

| 1. ("Pregnancy"[mh] OR "Maternal Exposure"[mh] OR "Prenatal Exposure Delayed Effects"[mh] OR "Maternal Nutritional Physiological Phenomena"[mh] OR pregnan*[tw] OR maternal [tw] OR prenatal[tw] OR antenatal [tw] OR perinatal [tw]) 2. ("Cohort Studies"[mh] OR "Prognosis"[mh] OR "Case-Control Studies"[mh] OR "Follow-Up Studies"[mh] OR cohort stud*[tw] OR case-control[tw] OR "Risk Factors"[mh] OR risk factor*[tw] OR "Clinical Trial" [pt] OR "Evaluation Studies" [pt] OR "Validation Studies" [pt] OR "Comparative Study" [pt] OR "Cross-Sectional Studies"[mh] OR random*[tw] OR cohort*[tw] OR risk*[tw] OR causa*[tw] OR predispos*[tw] OR odds ratio[mh] OR case control* OR odds ratio* OR controlled clinical trial [pt] OR randomized controlled trial [pt] OR risk[mh] OR practice guideline[pt] OR epidemiologic studies[mh] OR case control studies[mh] OR cohort studies[mh] OR age factors[mh] OR comorbidity[mh] OR epidemiologic factors[mh] OR clinical stud* [tw] OR research [tw] OR research stud* [tw]) 3. ("listeriosis"[mh] OR "listeria"[mh] OR listeri*[tw]) 4. "cheese"[mh] OR cheese*[tw] OR dairy products"[mh] or dairy*[tw] or "milk"[mh] OR milk*[tw] 5. "meat"[MeSH Terms] OR meat*[tiab] OR pate [tiab] OR chicken [tiab]OR salad* [tiab] OR unpasterui* [tiab] OR ice cream OR ice-cream [tiab] OR hot dog [tiab] OR “sandwiche*” [MeSh Terms] OR "fishes"[MeSH Terms] OR fish*[tiab] OR “seafood” [MeSH Terms] OR shellfish [tiab] OR prawns [tiab] OR “prawns” [MeSH] 6. "vegetables"[MeSH Terms] OR vegetable*[tiab] 7. “fruit” [MeSH Terms] OR fruit [tiab] 8. unpasteur*[tiab]) OR pasteur* [tiab] 9. food safety [tiab] OR hygiene [tiab] OR high risk food* [tiab] OR hygiene practice* [tiab] OR food quality [tiab] OR “hazard analysis critical control points” [tiab] OR food preparation [tiab] 10. #5 OR #6 OR #7 OR #8 OR #9 OR #10 11. #1 AND #2 AND #3 AND #11 |
| --- |

**Supplementary Table S2.** Characteristics of excluded studies

| **Author** | **Title** | **Reason for exclusion** |
| --- | --- | --- |
| Xu | Awareness of Listeria and high-risk food consumption behaviour among pregnant women in Louisiana | No reporting of listeriosis during pregnancy |
| Charlier | Clinical features and prognostic factors of listeriosis: the MONALISA national prospective cohort study | No association of listeriosis with food |
| Filipello | Epidemiology and Molecular Typing of Pregnancy-Associated Listeriosis Cases in Lombardy, Italy, over a 10-Year Period (2005–2014) | No association of listeriosis with food |
| Barkley | Preventing Foodborne and Enteric Illnesses Among At-Risk Populations in the United States and Rhode Island | No association of listeriosis with food |
| Gottlieb | Multistate outbreak of listeriosis linked to turkey deli meat and subsequent changes in US Regulatory Policy | Case-case comparison (outbreak vs sporadic) not case control |
| Goulet | Incidence of listeriosis and related mortality among groups at risk of acquiring listeriosis | No association of listeriosis with food |
| Silk | Infectious disease/CDC update. Vital signs: Listeria illnesses, deaths, and outbreaks-United States, 2009-2011 | No association of listeriosis with food |
| Pouillot | Relative risk of listeriosis in foodborne diseases active surveillance network (FoodNet) sites according to age, pregnancy, and ethnicity | No association of listeriosis with food |
| Aljicevic | Listeria monocytogenes as the possible cause of the spontaneous abortion in female of the fertile age | No association of listeriosis with food |
| Hussein | Occurrence of Listeria monocytogenes in poultry, fish & their products as well as its public health hazard on women | No association of listeriosis with food |
| Brent | Risk factors for listeriosis in Australia | Letter, not original research |
| Johnson | CDC National Health Report: leading causes of morbidity and mortality and associated behavioural risk and protective factors--United States, 2005-2013 | No case control design |
| Rocourt | Outbreak of Listeriosis in France, 1992 | Not in English |
| Zander | Listeriosis surveillance in Australia | Not original research |
| Rungan | Listeria--how much do pregnant women really understand about it? | No case control design or association of listeriosis with food |
| Latorre | Quantitative risk assessment of listeriosis due to consumption of raw milk | No case control design or association of listeriosis with food |
| Mangen | Cost-of-illness and disease burden of food-related pathogens in the Netherlands, 2011 | No case control design or association of listeriosis with food |
| Imanish | Estimating the attack rate of pregnancy-associated listeriosis during a large outbreak | No case control design |
| McLauchlin | A possible outbreak of listeriosis caused by an unusual strain of Listeria monocytogenes | No case control design |
| Havelaar | Disease burden of foodborne pathogens in the Netherlands, 2009 | No case control design |
| Gaulin | Challenges in listeriosis cluster and outbreak investigations, Province of Quebec, 1997-2011 | No case control design |
| Tappero | Reduction in the incidence of human listeriosis in the United States: Effectiveness of prevention efforts? | No case control design |
| Haghroosta | Isolation and identification of listeria monocytogenes in pregnant women in Ahvaz, Iran | No association of listeriosis with food |
| Not listed | Food-borne listeriosis in the United States | Summary of CDC update report 1992 |
| Not listed | Case-control study of listeriosis in England and Wales: progress report | Progress report only, no results |
| Fernandez Garayzabal | Occurrence of Listeria monocytogenes in raw milk | No assessment in pregnancy |
| Flight | Listeriosis in Auckland | No case control design |
| Not listed | Listeriosis in France | No case control design |
| Not listed | Listeriosis update | Summary of Goulet 1993 which was excluded due to not being in English |
| Kampelmacher | Listeriosis in humans and animals in the Netherlands (1958-1977) | No case control design or association of listeriosis with food |
| Sills | Epidemic perinatal listeriosis in Southern-California | No case control design, abstract only |
| Matyunas | The polar bar ice-cream recall – report exposure in pregnant women | No case control design |
| Devila | Foodborne disease in children emerges as public health issue | Not original research |
| Not listed | Human listeriosis, 1988 | No case control design |
| Bertrand | Diversity of listeria monocytogenes strains of clinical and food chain origins in Belgium between 1985 and 2014 | No case control design or association of listeriosis with food |
| Self | Outbreak of Listeriosis Associated with Consumption of Packaged Salad - United States and Canada, 2015-2016 | No case control design |
| Centers for Disease Control and Prevention | Notifiable Diseases and Mortality Tables 2015 | No case control design or association of listeriosis with food |
| Centers for Disease Control and Prevention | Notifiable Diseases and Mortality Tables 2016 | No case control design or association of listeriosis with food |
| Canadian Medical Association | Notifiable Diseases Annual Summary. 1998 | No case control design or association of listeriosis with food |
| Short | Laboratory-based surveillance of invasive Listeria monocytogenes in New Zealand | No case control design or association of listeriosis with food |
| Harte | Laboratory-based surveillance of listeriosis, 2010 | No case control design or association of listeriosis with food |
| Centers for Disease Control and Prevention | Final 2014 reports of nationally notifiable infectious diseases | No case control design or association of listeriosis with food |
| Centers for Disease Control and Prevention | Final 2012 reports of nationally notifiable infectious diseases | No case control design or association of listeriosis with food |
| Shallow | Incidence of foodborne illnesses -- FoodNet, 1997 | No case control design or association of listeriosis with food |
| Shallow | Incidence of foodborne illnesses: preliminary data from the Foodborne Diseases Active Surveillance Network (FoodNet) -- United States, 1998 | No case control design or association of listeriosis with food |
| Delaune | Outbreak of invasive listeriosis associated with the consumption of hog head cheese--Louisiana, 2010 | No case control design |
| Vugia | Preliminary FoodNet data on the incidence of infection with pathogens transmitted commonly through food--10 states, 2007 | No case control design or association of listeriosis with food |
| Olsen | Surveillance for foodborne-disease outbreaks -- United States, 1993-1997 | No case control design |
| Goulet | Listeriosis from consumption of raw-milk cheese | Doesn’t state if any pregnant women included in case-control study |
| McCollum | Multistate Outbreak of Listeriosis Associated with Cantaloupe | Case-case comparison (outbreak vs sporadic) not case control |
| Centers for Disease Control and Prevention | Multistate outbreak of listeriosis -- United States, 1998 | Doesn’t state if any pregnant women included in case-control study |
| Choi | Multistate Outbreak of Listeriosis Linked to Soft-Ripened Cheese - United States, 2013 | Case-case comparison (outbreak vs sporadic) not case control |
| Cosgrove | Multistate Outbreak of Listeriosis Associated with Jensen Farms Cantaloupe - United States, August-September 2011 | Case-case comparison (outbreak vs sporadic) not case control |
| Jackson | Multistate outbreak of listeria monocytogenes associated with Mexican-style cheese made from pasteurized milk among pregnant, Hispanic women | Case-case comparison (outbreak vs sporadic) not case control |
| Centers for Disease Control and Prevention | From the Centers for Disease Control and Prevention. Update: Multistate outbreak of listeriosis--United States, 1998-1999 | No case control design |
| Hurd | From the Centers for Disease Control and Prevention. Multistate outbreak of listeriosis--United States, 2000 | Doesn’t state if any pregnant women included in case-control study |
| Centers for Disease Control and Prevention | Update: multistate outbreak of listeriosis--United States, 1998-1999 | No case control design |
| Tahery | Listeria monocytogenesis and abortion: A case study of pregnant women in Iran | Case control for abortion vs no abortion rather than listeriosis vs non- listeriosis |
| Mead | Nationwide outbreak of listeriosis due to contaminated meat | Case-case comparison (two different strains of Listeria) not case control |
| Mascola | Fecal carriage of Listeria monocytogenes - Observations during a community-wide, common-source outbreak | Case control for stool carriage of listeriosis, not listeriosis itself |
| Silk | Vital signs: Listeria illnesses, deaths, and outbreaks - United States, 2009-2011 | No case control design |
| Groseclose | Summary of notifiable diseases -- United States, 2000 | No case control design or association of listeriosis with food |
| Groseclose | Summary of notifiable diseases -- United States, 2001 | No case control design or association of listeriosis with food |
| Groseclose | Summary of notifiable diseases -- United States, 2002 | No case control design or association of listeriosis with food |
| Hopkins | Summary of notifiable disease -- United States, 2003 | No case control design |
| Jajosky | Summary of notifiable disease -- United States, 2004 | No case control design |
| McNabb | Summary of notifiable diseases - United States, 2006 | No case control design |
| Pouillot | Quantitative risk assessment of Listeria monocytogenes in French cold-smoked salmon: II. Risk characterization | No case control design |
| de Valk | Two Consecutive Nationwide Outbreaks of Listeriosis in France, October 1999–February 2000 | Case-case comparison (outbreak vs sporadic) not case control |
| Olsen | Multistate Outbreak of Listeria monocytogenes Infection Linked to Delicatessen Turkey Meat | Case-case comparison (two different strains of Listeria) not case control |
| Lassen | Two listeria outbreaks caused by smoked fish consumption-using whole-genome sequencing for outbreak investigations. | Case-case comparison (outbreak vs non-outbreak) not case control |
| Lyytikäinen | Surveillance of listeriosis in Finland during 1995-2004. Euro Surveillance | Does not state if any pregnant women included in case-control study |

**Supplementary Table S3.** Detailed characteristics of included studies

| **Author** | **Cases - Recruitment source and method** | **Controls - Recruitment source and method** | **Matching of cases and controls** | **Definition of perinatal listeriosis** |
| --- | --- | --- | --- | --- |
| Schlech 1983 ^24^ | Acute-care hospital beds in Halifax or from other provinces | Same hospital | 4 controls per case  Two temporal (one born just before or after) at same hospital, two temporal and birth weight matched | Compatible clinical illness in a mother or child or both accompanied by a positive culture for L. monocytogenes from the mother, the child or placental surface |
| Fleming 1985 ^6^ | Survey of microbiological laboratories for reports of L. monocytogenes isolated from clinical specimens | Study 1: Town listings made available by the Harvard School of Public Health. Study 2: Infection-control practitioners where cases were hospitalised. For both studies cases with isolates of serotype other than the epidemic excluded | Study 1: 2 controls per case, age (within 10 years), sex, neighbourhood of residence. Study 2: One control per case, age (within 10 years) and primary underlying illness | L. monocytogenes isolated from placenta in conjunction with fetal death |
| Schwartz 1988 ^25^ | Doctors in surveillance area contacted for reports of all patients with positive sterile-site cultures  ICD-9 discharge diagnosis codes for L. monocytogenes reviewed from hospitals in surveillance areas | Doctor records or hospital infection control practitioner selected matched controls from recent hospital admissions | 4 controls per case  Age (+/- 10 years) and underlying disease. If case had more than one underlying conditions controls were matched for illness most likely to be immunosupressive | No details provided |
| Linnan 1988 ^21^ | First available maternal/neonatal patients  Review of medical records of all patients with listeriosis in Los Angeles County, clinical isolates submitted to California State Microbial Disease Laboratory and cases from health department officials; survey of infection-control practitioners and microbiologic laboratores at all acute-care hospitals | Identifying the next delivery after a case in the birth log of the case hospital that fit matching criteria | Hospital of delivery, age, sex, pregnancy status (gestational age) and ethnic group | L. monocytogenes isolated from culture of a normally sterile site of a pregnant woman, her neonate or fetus, or both |
| Schuchat 1992 ^27^ | Biweekly calls to infection control practitioners or designated contacts in microbiological laboratories serving all acute care hospitals in surveillance area; review of case report forms all patients in whom L. monocytogenes isolated from normally sterile site; periodic laboratory audits at hospital subset | Through cases obstetrician or hospital infection control practitioner identified from recent hospital admissions | 4 controls per case  Age (5 years), health care provider (obstetrician), approximate date of conception | L. monocytogenes isolated from case during pregnancy or perinatal period and/or from offspring within first 31 days of life |
| Jensen 1994 ^20^ | All laboratory-reported Danish cases | Same department and same hospital as case on the same date | Questionnaire period, geographical area of hospital administration, age and gender | Ill mother and unborn or newborn infant from whom L. monocytogenes was demonstrated |
| Goulet 1998 ^19^ | All hospital microbiology laboratories throughout France requested to send all human isolates of L. monocytogenes identified since 1 May 1993 to the National Reference Centre | Same hospital, selected by attending physicians or general practitioner of case-patient | 4 controls per case  Same gestational age (+/- 4 weeks) and attending same hospital | L. monocytogenes strain isolated from pregnant woman, neonate or fetus |
| MacDonald 2005 ^22^ | Female Hispanic residents of Winston-Salem, active case finding at the 2 hospitals in Forsyth County, North Carolina and contacting other regional hospitals and laboratories | County's prenatal care program registry and from office of Special Supplemental Nutrition Program for Women, Infants and Children' | 4 controls per case  Ethnicity, gender, age (within 1 year), pregnancy (trimester of pregnancy) | Experienced premature birth, stillbirth or febrile illness in association with isolation of L. monocytogenes from a normally sterile site or with placental tissue samples found to be positive for L. monocytogenes by staining performed using immunohistochemical techniques |
| Varma 2007 ^26^ | Active surveillance for confirmed cases of L. monocytogenes infection in >600 clinical diagnostic laboratories located in patient catchment area (mean population 37.7 million) including all or part of 9 US states  Data collected from FoodNet (Foodborn Diseases Active Surveillance Network)  Cases excluded if patient did not report illness, did not speak English or Spanish or acquired infection as part of a recognised outbreak | Physicians who treated case or from predetermined multispeciality clinic, health maintenance organisation or medical centre in FoodNet catchment area  Controls excluded if patient did not speak English or Spanish | 4 controls per case  1 month of estimated date of delivery | L. monocytogenes from a normally  sterile site, including from blood or CSF samples, or from placenta or the products of conception  If illness occurred in a pregnant women or an infant <31 days old |
| Dalton 2011 ^10^ | Identified from State and Territory health departments as listeriosis is a laboratory notifiable condition. | Perinatal controls: hospital-based antenatal clinics with gestational attainment within 4 weeks of matched case at time of positive culture. All controls recruited within 4 weeks of conducting case interview | Two controls per case  Age, primary underlying immunosupressive condition | Illness in pregnant woman, fetal loss, or illness in baby aged <3 months with isolation of L. monocytogenes from at least one of the woman, fetus or baby, or normally sterile site from a pregnant maternofetal pair from any of following sites: placenta or products of conception, fetal gastrointestinal contents or normally sterile site from pregnant woman, fetus or baby |
| Pourkaveh 2016 ^23^ | Pregnant women with spontaneous abortion complications. Women admitted to educational medical centres of Tehran, Iran with symptoms of spontaneous abortion such as bleeding, spotting and abdominal cramps; gestational age <20 weeks; no history of infertility; no methods of induced abortion and no ART techniques; non-smoking and/or using drugs; lack of chronic diseases such as diabetes and hypertension and abdominal trauma | Same for cases and controls | No matching as cross-sectional study with identical recruitment. Cases and controls significantly different for age, education, urban/rural residence, internet access | L. monocytogenes mucosal swabs of vaginal wall and cervix |

**Supplementary Table S4.** Quality appraisal of included studies

| **Author** | **Selection**  **Maximum 4 stars** | **Comparability**  **Maximum 2 stars** | **Exposures**  **Maximum 3 stars** | **Overall quality rating** |
| --- | --- | --- | --- | --- |
| Schlech 1983 ^24^ | 2 | 1 | 3 | Fair quality |
| Fleming 1985 ^6^ | 2 | 2 | 2 | Fair quality |
| Schwartz 1988 ^25^ | 3 | 1 | 3 | Good quality |
| Linnan 1988 ^21^ | 3 | 2 | 2 | Good quality |
| Schuchat 1992 ^27^ | 3 | 2 | 3 | Good quality |
| Jensen 1994 ^20^ | 2 | 2 | 2 | Fair quality |
| Goulet 1998 ^19^ | 2 | 1 | 2 | Fair quality |
| MacDonald 2005 ^22^ | 3 | 2 | 2 | Good quality |
| Varma 2007 ^26^ | 2 | 1 | 2 | Fair quality |
| Dalton 2011 ^10^ | 3 | 2 | 1 | Poor quality |
| Pourkaveh 2016 ^23^ | 1 | 0 | 1 | Poor quality |

SELECTION
(1) Is the case definition adequate? (a) yes, with independent validation*, (b) yes, e.g. record linkage or based on self-reports, (c) no description.
(2) Representativeness of the cases: (a) consecutive or obviously representative series of cases*, (b) not satisfying (a) or not stated.
(3) Selection of controls: (a) community controls*, (b) hospital controls, (c) no description.
(4) Definition of controls: (a) no history of disease (end-point)*, (b) no description of source.
COMPARABILITY
(1) Comparability of cases and controls on basis of design or analysis: (a) study controls for age*, (b) study controls for any additional factor*.
EXPOSURE
(1) Ascertainment of exposure: (a) secure record (e.g. surgical records)*, (b) structured interview where blind to case/control status*, (c) interview not blinded to case/control status, (d)
written self-report or medical record only, (e) no description.
(2) Same method of ascertainment for cases and controls: (a) yes*, (b) no.
(3) Non-response rate: (a) same rate for both groups*, (b) non-respondents described, (c) rate different and no designation
